# Clinical characteristics of children with Juvenile Systemic Sclerosis: follow-up of 23 patients in a single tertiary center

**DOI:** 10.1186/1546-0096-5-6

**Published:** 2007-05-01

**Authors:** Ricardo AG Russo, María M Katsicas

**Affiliations:** 1 Service of Immunology and Rheumatology, Hospital de Pediatría Prof. Dr. Juan P. Garrahan. Combate de los Pozos 1881, (1245) Buenos Aires, Argentina

## Abstract

**Background:**

Juvenile systemic sclerosis (JSS) is a multisystem connective tissue disease characterized by skin fibrosis and internal organ involvement. It has a low prevalence, even in a tertiary facility setting. The purpose of the present study is to describe and analyze the clinical and laboratory characteristics of a group of children with JSS followed in a single center.

**Methods:**

Clinical charts of children with a diagnosis of JSS who were seen at a tertiary referral center between 1995 and 2005 were reviewed. Clinical features were recorded and analysed.

**Results:**

Twenty-three patients who met preliminary classification criteria for JSS were included. Age at first symptom attributable to JSS was 6 (1–14) years, The first symptom attributable to JSS was Raynaud's phenomenon in 14 cases. Proximal sclerosis (23 patients, 100%), sclerodactyly (21, 91%), Raynaud's phenomenon (19, 83%), and periungual capillaropathy (17, 74%) were the most consistent clinical findings during follow-up. Respiratory involvement occurred in two thirds of our patients, and it manifested as dyspnea as well as abnormal imaging and/or pulmonary function tests; pulmonary hypertension was an infrequent finding. Dysphagia was the commonest gastrointestinal symptom (9 patients, 39%). The most frequent musculoskeletal symptom was arthralgia (14 children, 6%); symmetrical arthritis was found in 8 (35%) patients. Periungual capillary abnormalities were evident during physical examination in 17 children; capillaroscopy revealed abnormalities in all 19 examined patients. ANA were present in 17 (74%) children: homogeneous pattern was the most frequent (8 patients), nucleolar (5) and speckled (4) were less common.

**Conclusion:**

Raynaud's phenomenon heralds the beginning of the disease. Capilaroscopy is a major adjuvant in the diagnosis, since autoantibody determination may not offer sensitive and specific markers. Skin and vascular manifestations are the most common clinical features, while internal organ involvement is more rare. Cardiopulmonary disease is the most frequent visceral involvement, leading to significant morbidity.

## Background

Juvenile systemic sclerosis (JSS) is a multisystem connective tissue disease characterized by widespread skin fibrosis and internal organ involvement [1]. Recently, a new set of criteria was developed by an international consensus conference to allow the classification of children with JSS on the basis of clinical and laboratory parameters (Table 1). These preliminary criteria represent a major advance, since the classification criteria for adult Systemic Sclerosis of the American College of Rheumatology [2], which have been applied to children for decades, have not been validated in pediatric patients.

**Table 1:** Provisional classification criteria for the classification of Juvenile Systemic Sclerosis [8].

Major criterion (Required)	
Proximal (to MCP/MTP joints) skin sclerosis/induration of the skin	
Minor criteria (At least two required)	
Cutaneous	Sclerodactyly
Peripheral vascular	Raynaud's phenomenon
	Nailfold capillary abnormalities
	Digital tip ulcers
Gastrointestinal	Dysphagia
	Gastro-esophageal reflux
Cardiac	Arrhythmias
	Heart failure
Renal	Renal crisis
	New onset arterial hypertension
Respiratory	Pulmonary fibrosis (HRCT/radiograph)
	Decreased DLCO
	Pulmonary arterial hypertension
Neurologic	Neuropathy
	Carpal tunnel syndrome
Musculoskeletal	Tendon friction rubs
	Arthritis
	Myositis
Serologic	Antinuclear antibodies
	SSc selective autoantibodies
	(anticentromere, anti-topoisomerase I (Scl 70), anti-fibrillarin, anti-PM-Scl, anti-fibrillin or anti-RNA polymerase I or III)

JSS is rare condition that runs a chronic course. Single center-based reported series are small, revealing its low prevalence [3-7]. This fact has made the setting of large epidemiological and clinical studies difficult to achieve. The purpose of the present study is to describe and analyze the clinical and laboratory characteristics of a group of children with JSS followed in a single center.

## Materials and methods

The clinical charts of children with a diagnosis of JSS who were seen at Hospital de Pediatría "Prof. Dr. Juan P. Garrahan" (a tertiary referral center) between 1995 and 2005 were reviewed. Diagnosis was originally made on clinical grounds, on the basis of a combination of diffuse sclerodermatous skin changes and internal organ involvement, vascular abnormalities (including Raynaud's phenomenon) or presence of the following autoantibodies: antinuclear (ANA), anti-Scl-70 and anticentromere (ACA). Moreover, for the purpose of the present study, the new preliminary classification criteria for JSS [8] were applied to the patients. All patients who showed signs of another connective tissue disorder (such as Gottron's papules, heliotrope or malar rash) or met Kasukawa criteria for Mixed Connective Tissue Disease (MCTD) were excluded from this study.

The following demographic and clinical features occurring during the course of the disease were recorded: sex, age at first symptom related to JSS, age at diagnosis, presence of cutaneous (edema, sclerosis, sclerodactily, calcinosis, digital pitting, telangiectasias, skin thickening as measured by the Modified Rodnan Skin Score (MRS) [9]), cardiovascular (Raynaud's phenomenon, nailfold capillary changes, presence of pericarditis, hypertension, cardiac failure, arrhythmia, abnormalities of EKG, pulmonary hypertension as detected by Doppler echocardiography), respiratory (coughing, pleuritis, clinical and imaging signs of fibrosis, abnormalities in forced vital capacity and pulmonary diffusion capacity for carbon monoxide (DLCO), gastrointestinal (dysphagia, diarrhea, constipation, gastroesophageal reflux as evidenced by contrasted radiography or pH monitoring), musculoskeletal (arthritis, muscle involvement as defined by the presence of muscle weakness or elevation of muscle enzymes, tendon friction rubs, acrosteolysis), renal (proteinuria), and nonspecific (weight loss, growth retardation, eye dryness, seizures, peripheral neuropathy) symptoms. Presence of autoantibodies was also recorded: ANA (performed by indirect immunofluorescence (or IFI) on Hep II cells), anti Ro, anti La, anti RNP, anti Sm, anti Scl-70 (performed by immunoprecipitation), and ACA (IFI). Therapeutic approaches (use of corticosteroids, immunosuppressors and vasodilators) were also reviewed.

## Results

### Demographic and general features

Twenty-three patients were included. They all met provisional classification criteria for JSS: 19 patients met 3 or more minor criteria, 15 met 4 or more criteria, 12 met 5 or more criteria, and 5 children met 6 criteria. Median time of follow-up was 5 (1–11) years after their initial visit. Age at first symptom attributable to JSS was 6 (1–14) years, and duration of disease at first visit to our center was 1 (0.5 – 7) years. There was a marked predominance of girls (21) over boys (2). At the time of this report, only one patient of this cohort had died.

The first symptom attributable to JSS was Raynaud's phenomenon in 14 cases, skin induration in 7, and edema in 2 patients. Weight loss (weight below the third percentile) was observed in 15 children, and growth failure (height below the third percentile) was recorded in 3 patients. The most frequent clinical features are listed in Table 2.

**Table 2:** Frequency of symptoms during the course Juvenile Systemic Sclerosis

Symptom	# patients	(%)
Proximal sclerosis	23	(100)
Sclerodactily	21	(91)
Raynaud's phenomenon	19	(83)
Nailfold capillaries abnormalities	17	(74)
Digital pitting	15	(65)
Arthralgia	14	(61)
Edema	12	(52)
Calcinosis	11	(48)
Weight loss	11	(48)
Dysphagia	9	(39)
Arthritis	8	(35)
Muscle weakness	8	(35)
Dyspnea	6	(26)

No clinical finding could be associated to sex, age of onset or duration of the disease.

### Skin involvement

**Figure 1:**
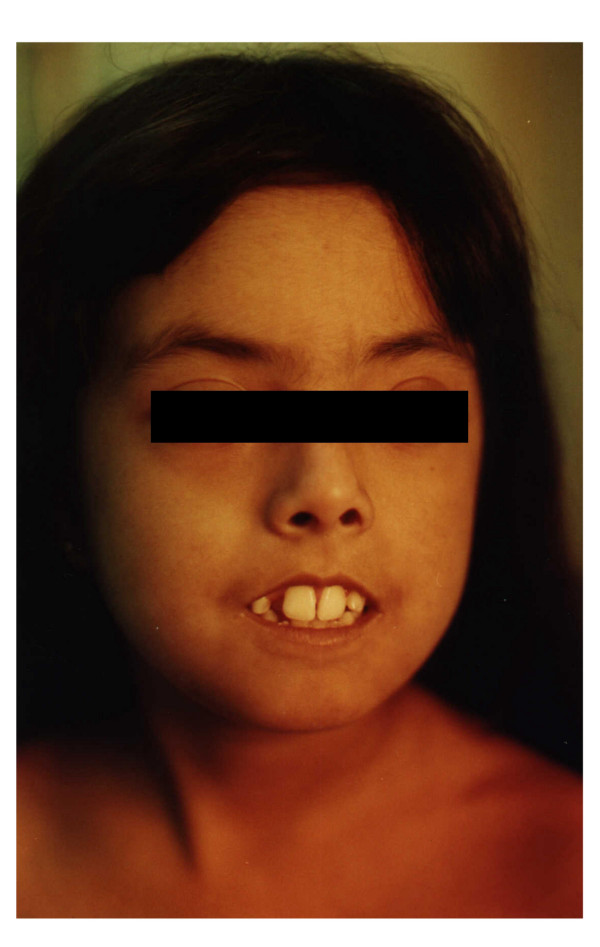
**Face of a 9 year-old girl with systemic sclerosis, 5 years after the disease onset**. Tightening of the skin is obvious. The patient is not able to close her lips.

**Figure 2:**
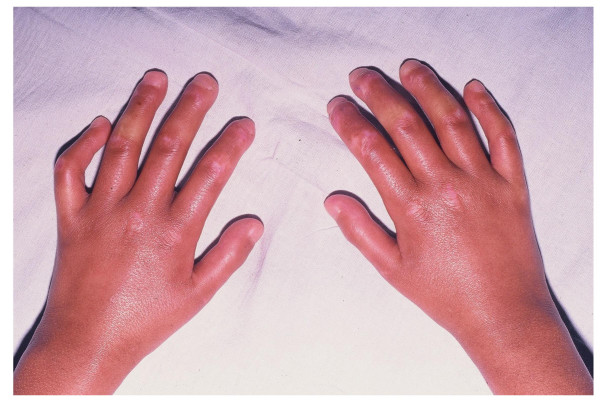
**Distal sclerosis in a patient with juvenile systemic sclerosis with disease duration of two years**. Skin is shiny and shows a light reddish discoloration over knuckles.

**Figure 3:**
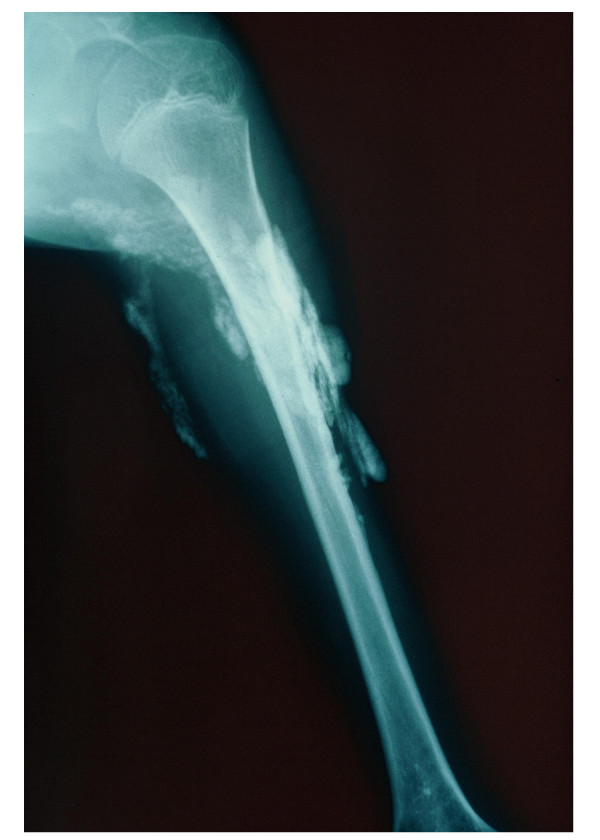
Proximal calcinosis in a patient with juvenile systemic sclerosis.

**Figure 4:**
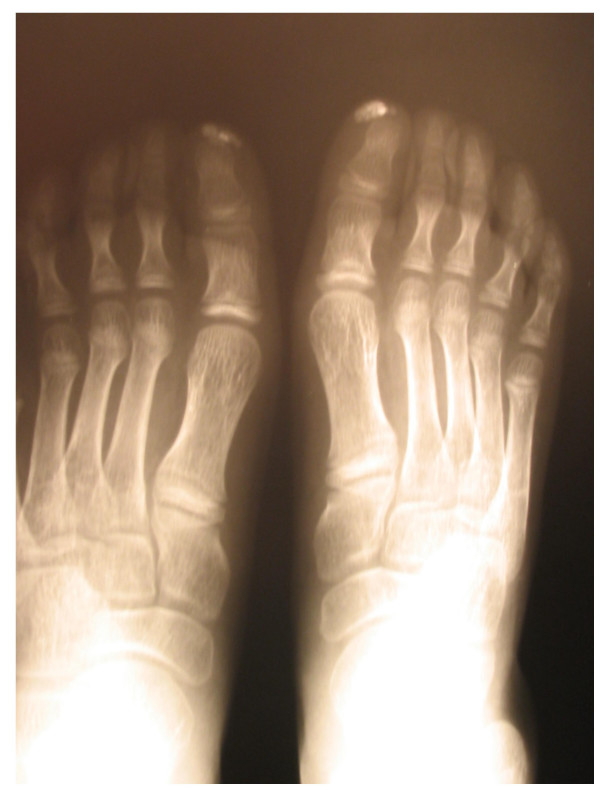
Distal calcinosis in a patient with juvenile systemic sclerosis.

All patients had skin induration proximal to the metacarpophalangeal and metatarsophalangeal joints (Figures 1 and 2); 21 (91%) children exhibited sclerodactyly; 11 (48%) patients showed evidence of calcinosis, which was located in fingers in 10 cases (Figures 3 and 4). Pitting in the finger pulps was present in 15 (65%) children; all of them had suffered from Raynaud's phenomenon for a variable length of time. Telangiectasias over the face, neck and trunk were recorded in 4 patients.

Periungual capillary abnormalities were evident during physical examination in 17 children; moreover, capillaroscopy revealed a wide range of abnormalities (Table 3) in all 19 examined patients: dilated loops and avascular areas were the most frequent. The classical scleroderma (or SD) pattern consisting in the simultaneous presence of megacapillaries and avascular areas [10] was present in 12 children. In those children in whom a thorough capillaroscopic follow-up was performed, there was a trend towards the progressive waning of megacapillaries and dilated loops and a growing frequency of avascular areas along the years.

**Table 3:** Main capillaroscopic findings in a cohort of patients with Juvenile Systemic Sclerosis.

Nailfold capillary abnormalities	# patients
Dilated loops	17
Avascular areas	16
Irregular loops	15
Hemorrhages	13
Megacapillaries	12
Dropout	12
Tortuousity	12
Arborization	11

**Figure 5:**
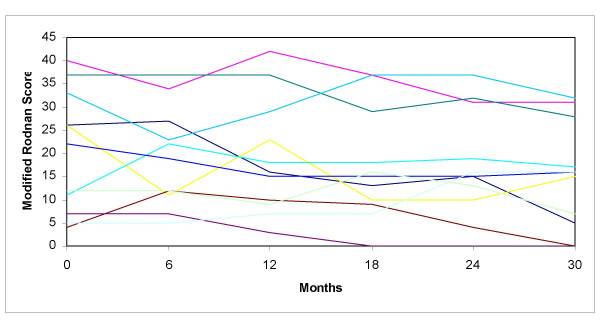
**Longitudinal skin thickness evaluation by Modified Rodnan Score in patients with Juvenile Systemic Sclerosis**. Each colour represents a patient.

Skin thickening was obvious in the first visit in all children: median MRS was initially 22 (4–40) and slowly decreased over the following years (to a median of 16 at 30 months after the first assessment) (Figure 5). MRS did not correlate with visceral involvement.

### Cardiovascular involvement

Raynaud's phenomenon was present in 19 (83%) children, and it was the initial symptom in 14 (61%). Two patients exhibited pericarditis, and in both cases it was low grade.

Arrhythmia was present in 2 girls: the first patient developed ventricular extrasystole, and she died due to this complication 7 years after disease onset, when she was 16 years old. The other girl had asymptomatic partial right bundle blockage.

Pulmonary hypertension was detected in 2 patients by means of abnormal echochardiographic findings. One of these patients also had evidence of pulmonary fibrosis.

### Respiratory involvement

Dyspnea was the most frequent respiratory complaint of children with JSS: it was present in 6 (26%) children. Dry coughing was less frequent (2 patients). Chest radiography evidenced pulmonary fibrosis in 9 children (manifested as bilateral basal interstitial infiltrates). Thoracic high resolution computed tomography (HRCT) was abnormal in 6 of 11 tested children, showing indirect signs of pulmonary fibrosis (reticular and ground glass opacification).

**Figure 6:**
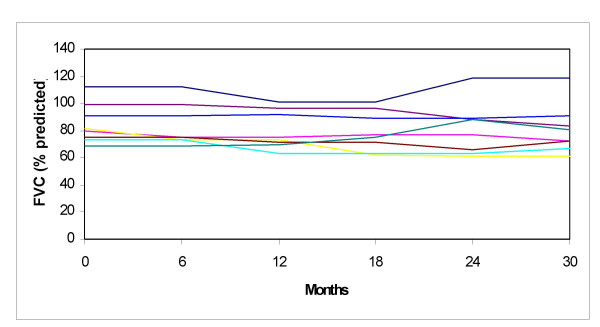
**Forced vital capacity (FVC) in a cohort of patients with Juvenile Systemic Sclerosis**. Tests were performed every six months. Patients with normal pulmonary imaging. Each colour represents a patient

**Figure 7:**
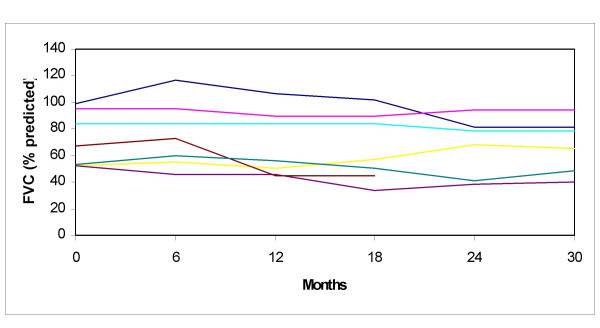
**Forced vital capacity (FVC) in a cohort of patients with Juvenile Systemic Sclerosis**. Tests were performed every six months. Patients with abnormal pulmonary radiography and/or computed tomography. Each colour represents a patient.

Pulmonary function tests (PFT) were abnormal in 15 (65%) children: they all exhibited restrictive incapacity (manifested as a forced vital capacity ≤ 80%) and in 3 of them DLCO was also decreased (≤ 80% of predicted normal). Seven of the 15 patients with abnormal PFTs did not show any abnormalities in pulmonary imaging. Patients with abnormal imaging tended to perform worse in FVC (Figures 6 and 7). Sequential PFTs showed that FVC and DLCO measurements did not show any meaningful changes over the years under heterogeneous therapeutic intervention. Evidence of small airway involvement, as detected by a decreased forced expiratory flow (FEF_25%–75%_), was present in 3 patients.

### Gastrointestinal involvement

Dysphagia was the commonest gastrointestinal symptom (9 patients, 39%). In 4 of these patients no abnormalities could be found in esophageal function. In the remaining 5 children, gastro esophageal reflux was present in 4 and esophageal dysmotility in 1. Constipation (4 children) and diarrhea (2 patients) were less frequent.

### Musculoskeletal involvement

The most frequent musculoskeletal symptom was arthralgia (14 children, 6%); symmetrical arthritis was found in 8 (35%) patients. Joint flexion contractures were common, especially over the interphalangeal joints of hands. Tendons "rubs" were recorded in 3 cases, usually over elbows and knees.

Eight children (35%) exhibited diffuse, symmetrical muscle weakness, but only 2 of them showed elevated serum muscle enzymes (creatinine kinase and aldolase).

Acro-osteolysis was found in 6 children who had suffered from a long-standing Raynaud's phenomenon,

### Renal involvement

Renal involvement was rare in this series. All patients had normal serum creatinine levels and urinalyses. However, 2 girls developed long-lasting, significant proteinuria (in the range of 500 to 5,000 mg/day). They underwent kidney biopsy, which revealed mesangeal proliferation in both, with no evidence of vascular, tubular or interstitial involvement. The proteinuria eventually disappeared over 2 to 5 years in both patients.

Scleroderma renal crisis did not occur in any of the patients.

### Other organic involvement

Xerophthalmia was found in 3 patients, who did not complain of xerostomia. One patient developed a transient episode of seizures. No peripheral neuropathy was detected.

Twelve (48%) children showed reduced weight for age, and height was below the 3^rd ^percentile for age in 3 children.

### Autoantibody profile

ANA were present in 17 (74%) children: homogeneous pattern was the most frequent (8 patients), while nucleolar (5) and speckled (4) were also found. ACA was only observed in 1 patient, who did not show any distinctive clinical manifestations. Anti-Scl-70 and anti-Ro antibodies were found in 2 patients each, while rheumatoid factor, anti-La and anti-RNP antibodies were revealed in one patient each. One patient with antinucleolar antibodies showed concomitant presence of anti-La, and another positive antinucleolar antibody child tested positive for anti-Scl 70.

None of these autoantibodies showed any correlation with clinical features.

### Pharmacologic treatment

All patients received an immunomodulator for varying length of time. Methotrexate (10–15 mg/m2/week, SQ) was used in 16 patients, D-penicillamine (up to 10 mg/Kg/day or 250 mg/day) in 12 children, and cyclophosphamide (Cyc, 500–1,000 mg/m2/month, for 6 months) in 2 patients who had progressive interstitial lung involvement. Systemic corticosteroids were used in 11 children, in all cases during the first year of the disease or in combination with Cyc. Vasodilators (nifedipine) were prescribed in 19 patients.

## Discussion

JSS is a rare disease. Its diagnosis may be difficult to make in its early stages, when edema and Raynaud's phenomenon predominate and skin induration may not be conspicuous. Early JSS might therefore resemble other conditions. This fact, along with the late development of pitting scars and abnormal radiography evidencing pulmonary fibrosis (criteria for the classification of adult patients with systemic sclerosis), and the overlap of JSS with other rheumatic diseases, lead to the formulation of classification criteria adapted to children, which will hopefully allow the appropriate inclusion of patients in clinical, epidemiological and basic research protocols [8].

The complexity of JSS usually determines the referral of potential or definite patients with the disease to tertiary centers, where a multidisciplinary approach is available. It is in such setting that we have been able to collect the clinical data of a small series of patients with JSS, which is certainly more numerous than most published series so far. In general, the frequency of clinical characteristics in our patients is similar to the one reported in the Padua database [11], of which most of our patients are constituents. In the present report, we deliberately excluded patients with overlap syndromes to obtain a more homogeneous group than the Padua cohort (which included patients with overlap syndrome and MCTD).

Most patients in our series were girls, and their debut occurred at a young age. Other series have also showed a female predominance, as well as later disease onset [3,5,7,12]. Patients with JSS in our series showed a wide range of clinical manifestations. Proximal sclerosis – which is a sine qua non condition for the diagnosis-sclerodactyly, Raynaud's phenomenon, telangiectasia, and presence of antinuclear antibodies were the most consistent findings. Visceral involvement was less frequent, and this fact might be related to the short duration of the disease in our group (median 6 years). However, at this moment no relationship between the development of internal organ involvement and duration of disease could be found.

Skin features in JSS are very similar to the ones observed in adult patients with systemic scleroderma. Raynaud's phenomenon usually precludes other findings, among which are the nailfold capillary changes. These show a wide spectrum of images, but the SD pattern is prevalent. Cutolo et al. have shown that changes in capillaroscopy reflect the different stages of the disease in patients with adult systemic sclerosis [13].

Skin induration slowly improved along the years, when pharmacological and physical therapeutic measures were used. Initially, it was moderate to high in most patients. Median MRS was 22, slightly higher than the one found by Scalapino et al. in their pediatric patients at their first visit (19.4) [12]. Patients with JSS usually develop a progressive atrophy of the skin, which may be associated with its thinning and a consequent lower MRS [1,14]. However, a recent study suggested that the use of the MRS needs to be validated in children, who show an increased score even in health, according to body mass index and Tanner stage [15]. For this reason, a MRS ≥ 14 should be considered a definite marker of skin thickening. As opposed to patients with the adult form of systemic sclerosis, no classification of patients based on the extent of skin induration could be made in our group, since all patients developed widespread, diffuse skin sclerosis, irrespective of their autoantibody profile.

Pulmonary disease is the most frequent of visceral involvement, usually related to pulmonary fibrosis. Respiratory involvement occurred in two thirds of our patients, and it manifested as dyspnea, abnormalities in imaging, and in PFTs. As it has been previously reported [3], radiographic changes correlated poorly with pulmonary function: about only half of our patients with abnormal FVC or DLCO showed abnormal imaging studies. Restrictive disease was the most common of spirometric patterns, while obstructive pattern with small airway involvement was found in 3 patients in our cohort. Similar findings have been previously reported by other investigators [6]. Pulmonary disease was not severe in most cases, but progressive fibrosis led to persistent restrictive incapacity – with significant impact on the patients' functional capacity- and abnormal pulmonary imaging in 2 children. These patients were treated with corticosteroids and IV cyclophosphamide for six months. After therapy PFTs seemed to stabilize over the following years. This therapeutic approach has proved efficacious in adult patients with systemic sclerosis. [16] On the other hand, all other patients did not show significant changes in their PFTs during follow-up. Garty et al. showed a similar behavior of PFTs in a followed cohort for 5 years [6].

Cardiovascular symptoms were not common in our group. In particular, pulmonary hypertension was an infrequent finding. The small number of patients with reduced DLCO reflects this fact. DLCO impairment is a marker of pulmonary vascular involvement, while decreased FVC usually indicates the presence of interstitial involvement [17]. Patients were treated with vasodilators, and at the time of this report pulmonary artery pressure is considered to be mildly elevated. Cardiac catheterization has not been performed in our patients

Although arrhythmia is an infrequent finding in children with JSS, it is a potentially severe complication that may be life-threatening, as evidenced by one of our patients. Heart disease has been the cause of death in several previously reported cases [3,6,18,19].

Esophageal dysfunction is the main cause of gastrointestinal morbidity in children with JSS [1]. It was present in 40% of our patients and it lead to significant discomfort. Dysphagia, diarrhea, intestinal involvement and anorexia may contribute to the marked weight loss observed in the majority of children. Since only radiography and pH monitoring were the methods used to detect gastroesophageal reflux, it may have been missed in some patients. Esophageal manometry, which was not performed in our patients, is a very sensitive method for detecting lower esophageal dysfunction [20].

As previously noticed in the report from the Padua database [11], arthralgia, arthritis and joint contractures were the most frequent musculoskeletal findings in our group. Myositis (proximal symmetrical muscle weakness accompanied by elevation of muscle enzymes) was observed in 2 patients. Similarly to previously reported cases of myositis in JSS [5,18], these patients exhibited restrictive pattern in PFTs and dysphagia due to esophageal dysfunction. However, none of them presented any myocardial involvement.

As opposed to adult patients with systemic scleroderma, "specific" autoantibodies are not common in the pediatric form of the disease [12,21]. Besides ANA – which occurred in the majority of patients- only 3 children showed antitopoisomerase-I (anti-Scl-70) or anticentromere antibodies, frequent markers of the disease in adults. On the other hand, the nucleolar pattern of the ANA was observed in 5 of our patients. Other investigators have reported this particular staining in children with JSS [5,22].

Pharmacological management of our patients included the use of immunomodulators. D-penicillamine was progressively replaced by methotrexate in the routine practice in our Service. In general, the appreciation of the efficacy of this overall therapy in our patients was considered to be satisfactory, in accordance with previous series [19]. One patient (out of 17 followed for at least 5 years) died. The resultant 5-year survival for our group is 94%, which coincides with previously reported survival rates [19] and seems to be better than the survival of patients in previous decades [4].

## Conclusion

JSS is a low-prevalence disease and incidental cases are very sporadic, even in a tertiary facility setting (2.3 new cases/year in our center). This is, to the best of our knowledge, the largest single-center based JSS cohort reported so far. Raynaud's phenomenon heralds the beginning of the disease. Capillaroscopy is a major adjuvant in the diagnosis, since autoantibody determination may not offer sensitive and specific markers. Skin and vascular manifestations are the most common clinical features, while internal organ involvement is more rare. However, cardiopulmonary disease is the most frequent visceral involvement, leading to significant morbidity, and potentially death. Prospective, multicentric studies with longer follow-up are needed to assess the natural history of the disease, survival, and provide the clinical basis for eventual clinical trials in the future.
